# Epigenetic aging studies of pair bonding in prairie voles

**DOI:** 10.1038/s41598-024-67641-2

**Published:** 2024-07-29

**Authors:** Lindsay L. Sailer, Amin Haghani, Joseph A. Zoller, Caesar Z. Li, Alexander G. Ophir, Steve Horvath

**Affiliations:** 1 Department of Psychology, Cornell University, Ithaca, NY 14853 USA; 2 Altos Labs, San Diego, USA; 3 Department of Biostatistics, School of Public Health, University of California, Los Angeles, CA USA; 4 Department of Human Genetics, David Geffen School of Medicine, University of California, Los Angeles, CA USA

**Keywords:** Vole, Sex-naive, Bonding, Monogamy, Epigenetic clock, DNA methylation, Social evolution, Ageing, Epigenetic memory

## Abstract

The quality of romantic relationships can predict health consequences related to aging. DNA methylation-based biomarkers of aging accurately estimate chronological age. We developed several highly accurate epigenetic aging clocks, based on highly conserved mammalian CpGs, for the socially monogamous prairie vole (*Microtus ochrogaster*). In addition, our dual-species human-vole clock accurately measured relative age and illustrates high species conservation of epigenetic aging effects. Next, we assessed how pair bonding impacts epigenetic aging. We did not find evidence that pair-bonded voles exhibit accelerated or decelerated epigenetic aging effects in blood, ear, liver, or brain tissue. Our epigenome wide association study identified CpGs in five genes strongly associated with pair bonding: *Foxp4*, *Phf2, Mms22l, Foxb1,* and *Eif1ad*. Overall, we present accurate DNA methylation-based estimators of age for a species of great interest to researchers studying monogamy in animals. We did not find any evidence that sex-naive animals age differently from pair-bonded animals.

## Introduction

A significant aspect of human nature is forming social relationships between family members, friends, and romantic partners. High-quality social relationships are frequently thought to be powerful predictors of long-term health, well-being, and lifespan^1^. Marriage is perhaps the most obvious social relationship among humans, and the quality of marital relationships is known to moderate health outcomes and emotional well-being, sometimes in a sex-specific manner^2–5^. In addition to cardiovascular disease, poor marital quality is related to poor health, anxiety and/or depression, and negative family interactions predict biobehavioral reactivity for anxiety, depression, and allostatic load^6,7^. Marital dysfunction is associated with higher rates of poor diet and lower rates of physical activity, which over time degrades physical health and can help explain the mechanisms by which social relationships impact health^8^. The strength of social relationships is a predictor of the risk of death^9^. Thus, longevity and health across the lifespan are clearly susceptible to social support and the quality of social bonds in some way.

Despite the persuasive evidence consistent with the idea that social relationships impact aging and lifespan, conducting empirical studies providing direct support or causal evidence for this belief is challenging. Rodent models have been of great use in studies focused on longevity and healthy aging because they have significantly shorter lifespans in comparison to humans. However, most rodents lack the propensity to form pair bonds, a key feature that is so definitive of humans^10^ and relatively rare among mammals in general^11^. Prairie voles (*Microtus ochrogaster*), on the other hand, are an excellent model species for investigating the neurobiology of complex social behaviors because they form long-term socially monogamous bonds with their mates^12,13^, and both parents exert significant and relatively equal effort to raise their young^14,15^. In laboratory settings, prairie voles that cohabitate and mate with an opposite-sex partner for an extended period (> 24 h) demonstrate behavior that is consistent with a pair bond^16^, including a robust preference for their partner over a stranger and selective aggression toward unfamiliar intruders, whereas sex-naive individuals do not demonstrate these behaviors. Moreover, prairie voles that have lost a mate show depressive-like behaviors consistent with ‘grief’ and display impaired pair bond-related behaviors^17^. The life expectancy of prairie voles in the wild ranges between 4 to 7 months, due to factors such as population density, season of birth, and natal social group structure^18,19^, whereas laboratory-reared prairie voles typically live up to 1–2 and on very rare occasions may reach 3+ years^20–22^. Taken together, the relatively short lifespan of prairie voles and their complex social behaviors provide a rich opportunity to examine the effects of social bonds on longevity, aging, and lifespan^23^.

Although certain epigenetic mechanisms have recently been studied in the context of pair bonding and parental care in prairie voles^24–28^, the impact of pair bonding on age-related epigenetic changes is an important consideration that has not received attention until now. DNA methylation (DNAm), the most studied epigenetic modification, chiefly occurs on cytosines followed by guanine residues (CpG) along the 5ʹ→3ʹ direction^29^. DNAm plays an essential role in various developmental and genomic contexts^30–32^. Methylation of CpGs is dynamic during development and is known to regulate experience-dependent changes, such as those resulting from early-life adversity and reward-related experiences. For example, DNAm can fine-tune neuronal gene expression and social behavior outcomes in response to the type of parental care received during early postnatal development^28^.

Growing evidence has suggested that epigenetic markers of aging based on DNA methylation data can accurately estimate chronological age for any tissue across the entire lifespan of mammals^33–39^. These DNAm-based age estimators, also known as epigenetic clocks, target dozens to hundreds of aging-related CpG loci and apply penalized regression models to predict chronological age based on DNA methylation levels (reviewed in^39^). By examining various mammalian tissues and cell types, a robust correlation between chronological age and DNAm age over the course of entire lifespans has been well established with the human pan tissue DNAm age estimator^33^. Similar pan tissue clocks have been established in mice and many other mammals^37,38,40–46^.

In the present study, we used multiple tissues (blood, brain, ear, and liver) from pair-bonded and sex-naive (unpaired) prairie voles, of both sexes and across a wide range of ages, to develop a highly accurate pan-tissue prairie vole DNAm clock for relating DNAm age with chronological age across multiple tissues. We next assessed the degree to which the prairie vole DNAm clock is conserved, by comparing it to the human DNAm clock to assess the potential translatability of the two. Because of the potential health benefits associated with healthy bonded relationships, we then turned our focus to determine if remaining single impacts epigenetic aging at a different rate than being pair-bonded (across and within specific tissues). Finally, we performed epigenome-wide association studies (EWAS) to assess the degree to which specific genes show differential epigenetic modification as a response to pair bonding status.

## Results

### Data sets

We used the mammalian methylation array (HorvathMammalMethylChip40^47^) to generate pan-tissue and tissue-specific prairie vole epigenetic clocks from 330 samples from four different tissues of male and female prairie voles (blood, brain, ear, and liver, Table 1). The ages of the male and female prairie voles ranged from 0.063 to 1.31 years old (Table 1). Unsupervised hierarchical clustering of the methylation data revealed that the samples clustered by tissue type and sex (Supplementary Fig. 1). Pair bonding status did not appear to correspond to distinct clusters. Additionally, we used DNA methylation profiles from 1366 human samples, from several tissues and with a large age range, to construct two dual species human-vole epigenetic clocks. These human data were generated on the same custom methylation array, which was designed to facilitate cross-species comparisons across mammals.

**Table 1:** Description of the data.

Tissue	N	No. female	Mean age	Min. age	Max. age
Blood	48	23	0.611	0.11	1.31
Brain	95	48	0.582	0.063	1.31
Ear	95	48	0.584	0.063	1.31
Liver	92	46	0.573	0.063	1.31

N = Total number of tissues. Number of females. Age (years): mean, minimum and maximum.

### Epigenetic clocks

**Figure 1:**
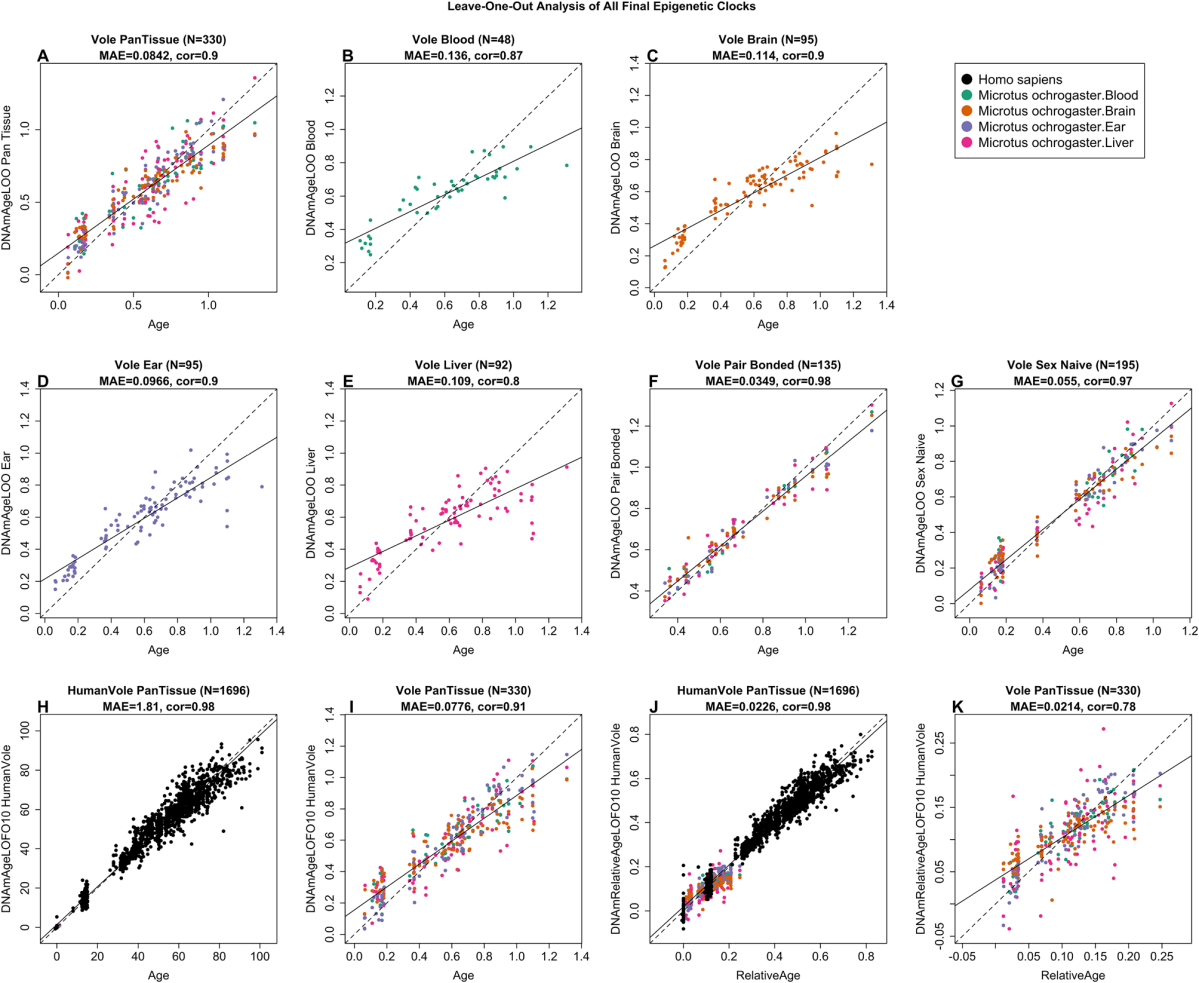
Cross-validation study of epigenetic clocks for prairie voles and humans. Leave-one-sample-out (LOO) estimate of DNA methylation age (y-axis, in units of years) versus chronological age. (**A**) Vole pan-tissue clock derived from all tissues (blood, brain, ear, and liver). Same clock as in panel A but restricted to vole blood samples (**B**), brain samples (**C**), ear samples (**D**), and liver samples (**E**). (**F**) Vole pair-bonded clock derived from all tissues of pair-bonded subjects. (**G**) Vole sex-naive clock derived from all tissues of sex-naive subjects. (**H**) Ten-fold cross-validation analysis of the human-vole clock for absolute age. (**I**) Same clock as in pnel H but restricted to voles. (**J**) Ten fold cross validation analysis of the human-vole clock for relative age, which is the ratio of chronological age to the maximum lifespan of the respective species. (**K**) Same clock as in panel **J** but restricted to voles. Dots are colored by tissue type (black = human tissue, green = vole blood, orange = vole brain tissue, purple = vole ear tissue, pink = vole liver tissue). Each panel reports the sample size, correlation coefficient, median absolute error (MAE). The black solid and dashed lines correspond to the regression line and the diagonal reference line (y = x), respectively.

We performed a cross-validation study in the training data to attain unbiased estimates of the age correlation *R* (defined as the Pearson correlation) between DNAm age (i.e., estimated age) and chronological age. The median absolute error (MAE, in units of years) was also calculated, which indicates concordance of the DNAm age with chronological age. Our different clocks can be distinguished by two dimensions: species and measure of age. The vole pan-tissue clock applies to multiple tissues (N = 330) from prairie voles, with a high age correlation of *R* = 0.90 and MAE of 0.084 between chronological age and DNAm age (Fig. 1A). We also defined tissue-specific clocks for voles with high age correlations: blood clock (R = 0.87, MAE = 0.136, N = 48; Fig. 1B), brain clock (R = 0.90, MAE = 0.114, N = 95; Fig. 1C), ear clock (R = 0.90, MAE = 0.097, N = 95; Fig. 1D), and liver clock (R = 0.80, MAE = 0.109, N = 92; Fig. 1E). Interestingly, we observed a higher age correlation and lower median absolute error in the vole pair-bonded clock (R = 0.98, MAE = 0.035, N = 135; Fig. 1F) and the vole sex-naive clock (R = 0.97, MAE = 0.055, N = 195; Fig. 1G), when we restricted samples by pair bonding status and not by tissue type. This could indicate that the vole pair-bonded clock provides a more accurate estimation of DNAm age over chronological age, however, fewer samples and more representation of adult ages are also possible driving factors of better performance.

**Figure 2:**
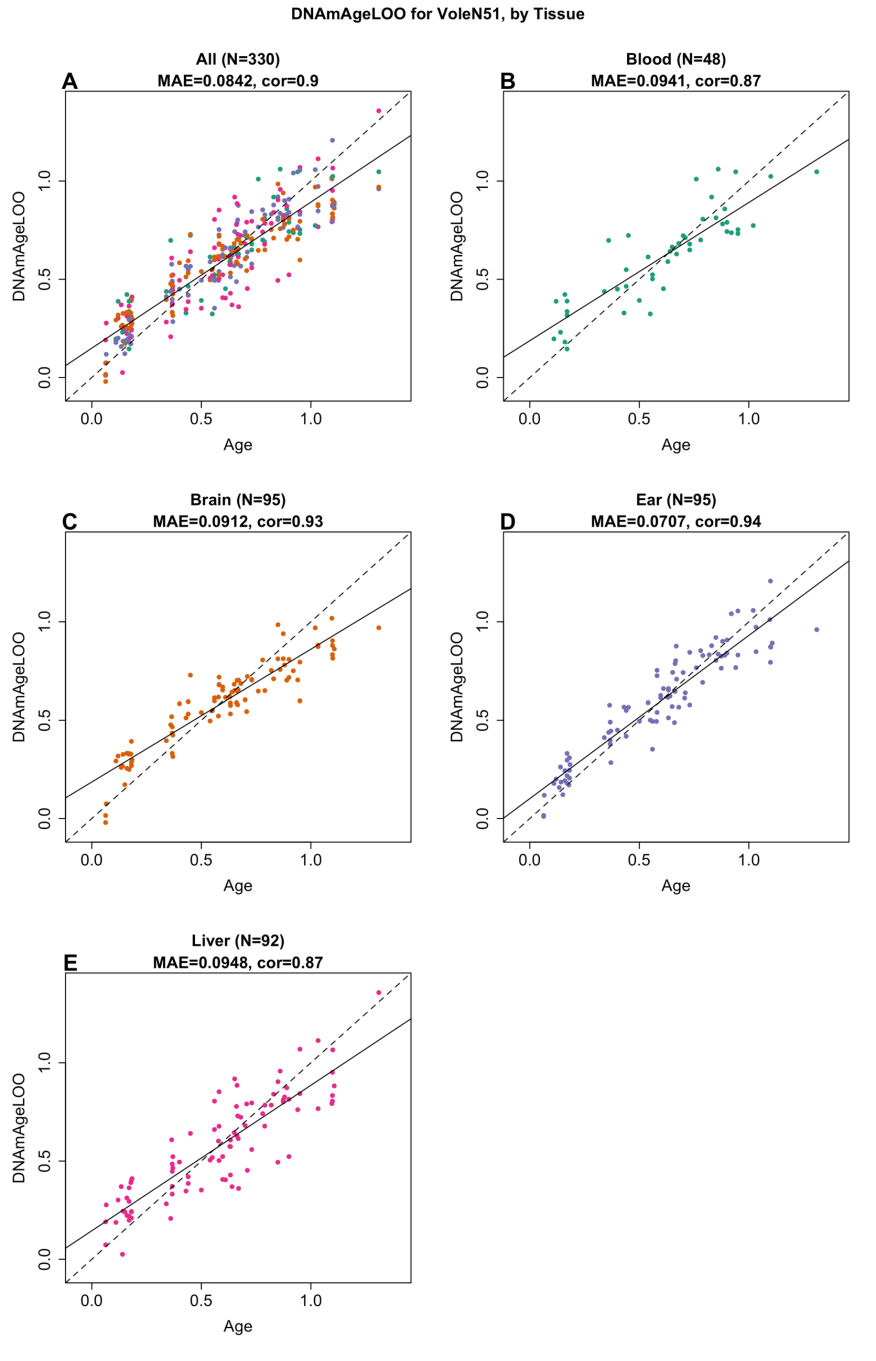
The multi-tissue epigenetic clock for prairie voles. Leave-one-sample-out (LOO) estimate of age based on DNA methylation data (x-axis) versus chronological age (in units of years) for (**A**) all tissues, (**B**) blood, (**C**) brain, (**D**) ear, (**E**) liver. Each panel reports the sample size, Pearson correlation coefficient and median absolute error (MAE). The black solid and dashed lines correspond to the regression line and the diagonal reference line (y = x), respectively.

The human-vole pan-tissue clock for chronological age can be used to estimate the chronological age of humans and voles using the same mathematical formula. The human-vole clock exhibits a high age correlation across both species (R = 0.98, MAE = 1.81, N = 1696; Fig. 1H) and maintains a strong performance when restricted to samples from voles (R = 0.91, MAE = 0.078, N = 330 Fig. 1I). The human-vole pan-tissue clock for *relative age*, defined as the ratio of chronological age to maximum lifespan, exhibits a high relative age correlation across both species clock (R = 0.98, MAE = 0.023, N = 1696; Fig. 1J) and a moderate performance was observed when restricted to samples from voles (R = 0.78, MAE = 0.021, N = 330; Fig. 1K). By definition, the relative age takes values between 0 and 1 and arguably provides a biologically meaningful comparison between species with different lifespans (vole and human), which is not afforded by the mere measurement of absolute age. When we restricted samples by tissue type, the vole pan-tissue clock maintained a high age correlation for the different tissue samples (Figs. 1A and 2A): cross validation in blood (R = 0.87, MAE = 0.094, N = 48; Fig. 2B), in brain (R = 0.93, MAE = 0.091, N = 95; Fig. 2C), in ear (R = 0.94, MAE = 0.071, N = 95; Fig. 2D), and in liver (R = 0.87, MAE = 0.095, N = 92; Fig. 2E) samples.

### Epigenetic clock analysis of pair bonding status

Our investigation set out to explore whether a relationship exists between pair bonding status (pair-bonded versus sex-naive) and epigenetic age acceleration in different tissues of voles. In this experiment, we housed sex-naive voles with a same-sex sibling, thereby ensuring they were not subjected to social isolation, unlike the pair-bonded animals who cohabited with a partner. However, controlling for sexual experience was not feasible as housing sexually experienced voles with a conspecific other than their mating partner induces elevated aggression. Our analysis included only tissues from sexually mature voles, namely 135 pair-bonded and 123 sex-naïve voles, all older than 0.3 years.

To investigate whether tissue samples from adult sex-naïve animals exhibit different epigenetic aging patterns compared to those from pair-bonded animals, we used several epigenetic clocks. First, we used the vole-specific pan-tissue clock. However, it did not find any significant evidence suggesting that tissues from adult sex-naive voles (age > 0.3 years) were epigenetically older than their pair-bonded counterparts (Wald test p > 0.5 from a multivariate regression model).

Second, we evaluated tissue-specific clocks (blood, brain, liver, and ear) and found again no significant association in multivariate regression models.

Third, we implemented two supplementary clocks: one pan-tissue clock exclusively for sex-naive voles, named the 'sex-naïve clock', and another solely for pair-bonded voles named the 'pair-bonded clock'. Both clocks demonstrated strong age correlations with the vole pair-bonded clock (R = 0.98, MAE = 0.035, N = 135; Fig. 1F) and the vole sex-naive clock (R = 0.97, MAE = 0.055, N = 195; Fig. 1G).

**Figure 3:**
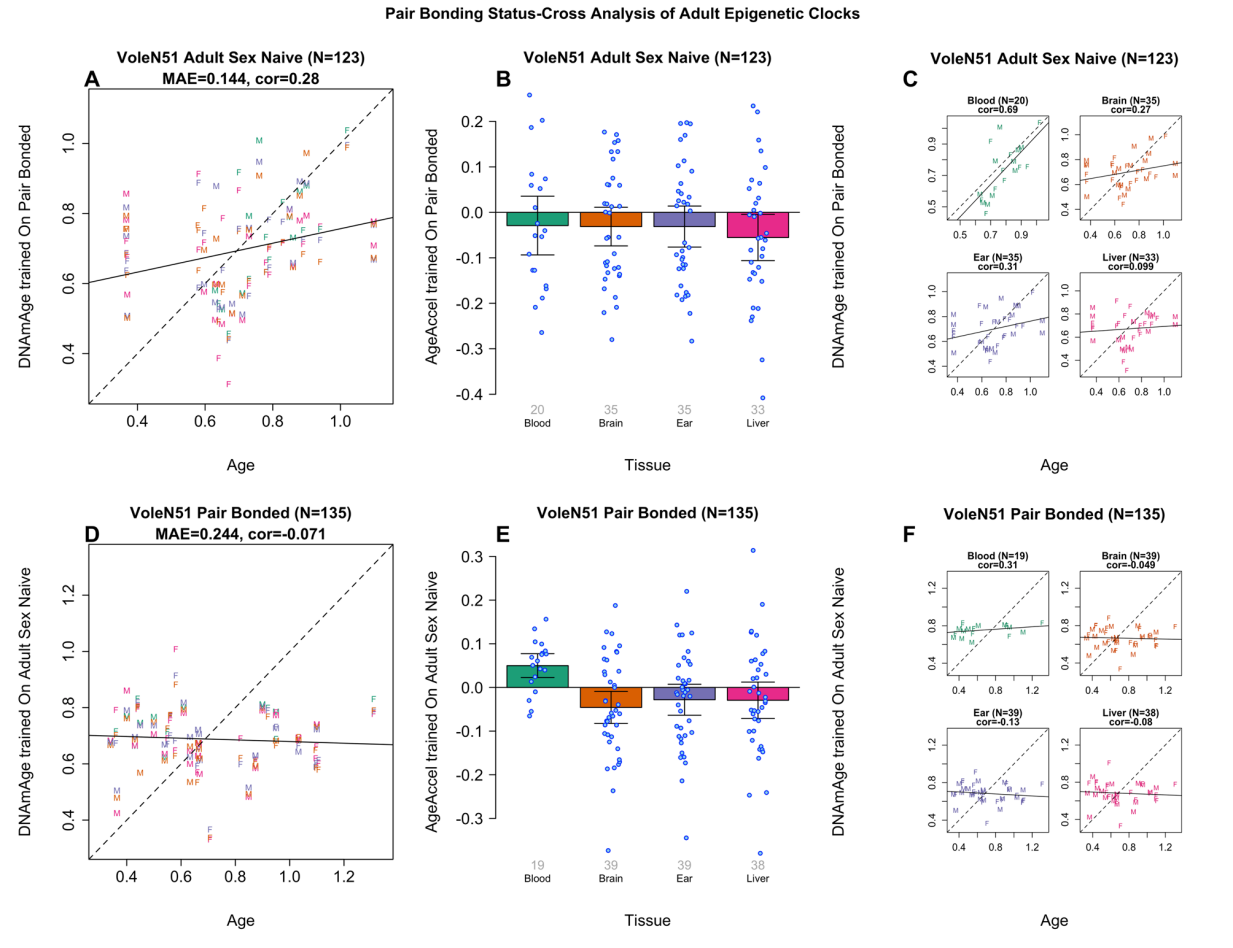
Epigenetic clock analysis of pair bonding status in brain samples. Age of adult sex-naive animals (x-axis) versus the age estimate according to the pan-tissue vole clock trained on pair-bonded animals in (**A**) all tissues combined, (**C**) individual tissues (blood, brain, ear, and liver). Age of adult pair-bonded animals (x-axis) versus the age estimate according to the pan-tissue vole clock trained on sex-naive animals (y-axis) in (**D**) all tissues combined, (**F**) individual tissues. Dots are colored by tissue type: green = blood, orange = brain, purple = ear, pink = liver. Samples are labelled by sex (F = female, M = male). (**B**,**E**) Age adjusted estimates of the cross validated age estimates versus tissue. The error bars depict two standard errors, so if they cross over the 0-line, then the two-sided p-value is greater than 0.05. All voles younger than 0.3 years were sex-naive and were therefore restricted from these analyses. For panels (**A**), (**C**), (**D**), and (**F**), the black solid and dashed lines correspond to the regression line and the diagonal reference line (y = x), respectively.

By definition, pair-bonded voles must be sexually mature (age > 0.3 years), whereas sex-naive voles can be any age. Some of our sex-naive samples were from juvenile voles, younger than the age of sexual maturity, raising concerns about potential extrapolation errors when applying the pair-bonded clocks. To mitigate this, we used the pair-bonded clock on adult sex-naive voles (age > 0.3 years, Fig. 3A–C) and vice versa (Fig. 3D–F). When applied to sex naïve animals, the pair-bonded clock showed limited accuracy (Pearson correlation R = 0.28, MAE = 0.144, N = 123; Fig. 3A). Conversely, the sex-naïve clock, when applied to pair bonded animals, exhibited a low correlation (R = 0.07, MAE = 0.244, N = 135, Fig. 3).

We utilized these clocks to test the hypothesis that tissues from sex-naive animals are epigenetically older than those from pair bonded animals. Contrary to expectations, the pair-bonded clock did not overestimate the age of tissues from sex naïve animals (Fig. 3B). In fact, the opposite was observed (one-sample t-test; blood t(19) = 0.899, p = 1.472; brain t(34) = 1.472, p = 0.150; ear t(34) = 1.388, p = 0.174; liver t(32) = 2.175, p = 0.037; Fig. 3B). Similarly, the sex -naïve clock, when applied to pair bonded animals, did not consistently lead to underestimation. Instead, it indicated that the epigenetic age of blood samples was older than expected (Fig. 3E). According to the sex-naïve clock, pair-bonded age acceleration was significantly faster in blood samples, significantly slower in brain samples, but not significantly different in ear and liver samples (one-sample t-test; blood t(18) = 3.650, p = 0.002; brain t(38) = 2.493, p = 0.017; ear t(38) = 1.590, p = 0.120; liver t(37) = 1.410, p = 0.167; Fig. 3E). These findings do not support the initial hypothesis.

In testing the alternative hypothesis that sex-naive animals are epigenetically younger, the pair-bonded clock applied to sex-naive animals did lead to an underestimation (Fig. 3B). However, the sex-naive clock applied to pair-bonded animals resulted in an overestimation for blood but not for the other tissues (Fig. 3E). This analysis is limited by the low accuracy of the sex-naive clock (Fig. 3D,F), rendering the results arguably inconclusive. Overall, the evidence is ambiguous and does not conclusively support either hypothesis regarding the epigenetic aging of sex-naive versus pair-bonded animals.

### EWAS of age

**Figure 4:**
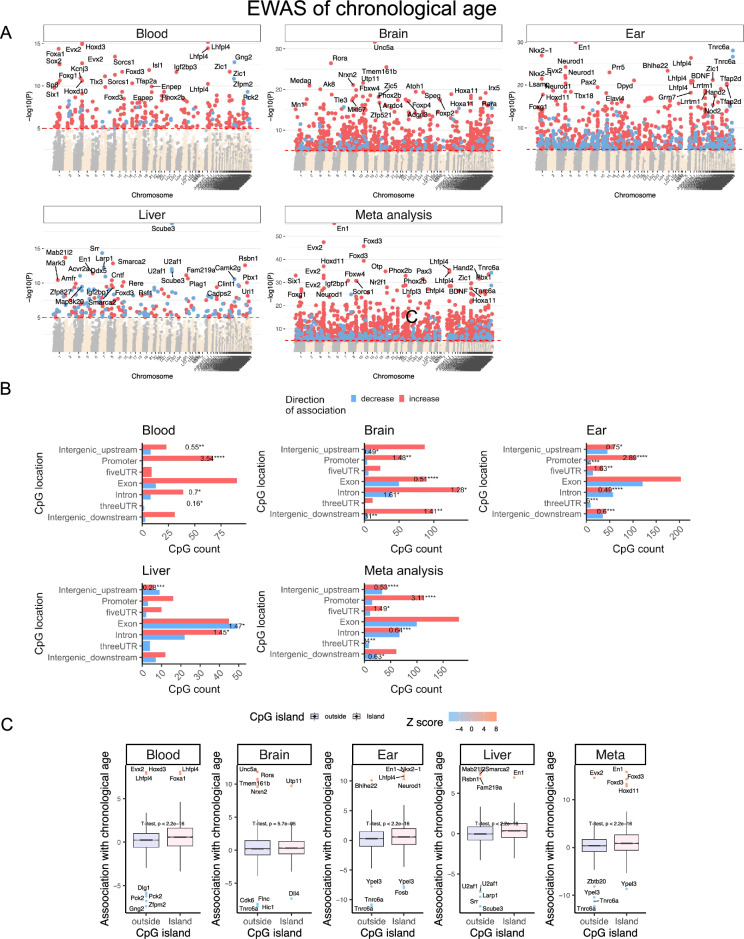
Epigenome-wide association study of age in tissues from prairie voles (*Microtus ochrogaster*). (**A**) Manhattan plots of the EWAS of chronological age. The coordinates are estimated based on the alignment of Mammalian array probes to MicOch1.0.100 genome assembly. CpG with a significant correlation with age (nominal, two-sided Pearson correlation test *p* < 10^–5^, red dotted line) are colored in red (age related gain of methylation) and blue (loss of methylation). The top 15 most significant CpGs are labeled by the neighboring genes. (**B**) Location of top CpGs in each tissue relative to the closest transcriptional start site. The odds ratio of the proportion changes than the background is reported in for each bar. Fisher exact p values: *p < 0.05, **p < 0.01, ***p < 0.001, ****p < 0.0001. The number of selected CpGs: blood, 295; brain, 614; ear, 786; liver, 226; meta-analysis, 759. (**C**) box plots of aging effects on CpGs (y-axis) versus CpG island status. The y-axis is the Fisher transformed z-score of the Pearson correlation of each CpG with age. The Student t-test *p* values are labeled above the box plots.

EWAS studies have the potential to identify markers within the genome that can be responsible for phenotypic variation. In total, 33,056 probes from HorvathMammalMethylChip40 were aligned to specific loci approximate to 5,210 genes in the prairie vole genome (*Microtus ochrogaster,* MicOch1.0.100). These probes have high conservation with human and other mammalian genomes. Epigenome-wide association studies of chronological age revealed a tissue-specific DNAm change in the prairie voles (Fig. 4A).

Methylation increases associated with aging are evident within gene promoter regions in blood, brain, and ear tissues (Fig. 4B). Additionally, CpG islands demonstrate a significant age-related increase in methylation levels when compared to genomic regions outside of CpG islands (Fig. 4C). The aging effects in one tissue seem to be poorly conserved in another tissue (Supplementary Fig. 2). However, the poor conservation and differences in *p*-value ranges in our analyzed tissue types may reflect a limited sample size in non-blood tissues. To capture the top affected loci in all tissues, DNAm was studied at a nominal *p*-value < 10^–5^. The top DNAm changes and the proximate genic region in each tissue were as follows: blood, Hoxd3 intron (z = 8.0); brain, *Unc5a* exon (z = 11.85); ear, *En1* promoter (z = 11.5); and liver, *Scube3* exon (z = − 9.0). In the meta-analysis of these three tissue samples, the top DNAm changes included hypermethylation in *En1* promoter (z = 15.8)*, Evx2* downstream (z = 14.5), *Foxd3* exon (z = 14.3), and hypomethylation in *Tnrc6a* exon (z = − 12.3). The genes implicated by our EWAS of age are enriched a wide range of biological processes related to development (e.g., skeletal system development), and metabolism particularly in liver (e.g., serine metabolism) (Supplementary Fig. 3). Moreover, aging-associated increase in methylation occurs at locations marked by H3K27me3, polycomb repressive complex 2 (PRC2) target sites, and bivalent regulatory regions (BivProm2) (Supplementary Fig. 4). Polycomb repressive proteins regulate H3K27Me3 marks, DNA damage, and senescence states of the cells during aging^48^. Our findings are consistent with previous work revealing that CpGs in PRC2 target sites *gain* methylation with aging^49^.

### EWAS of pair bonding status

**Figure 5:**
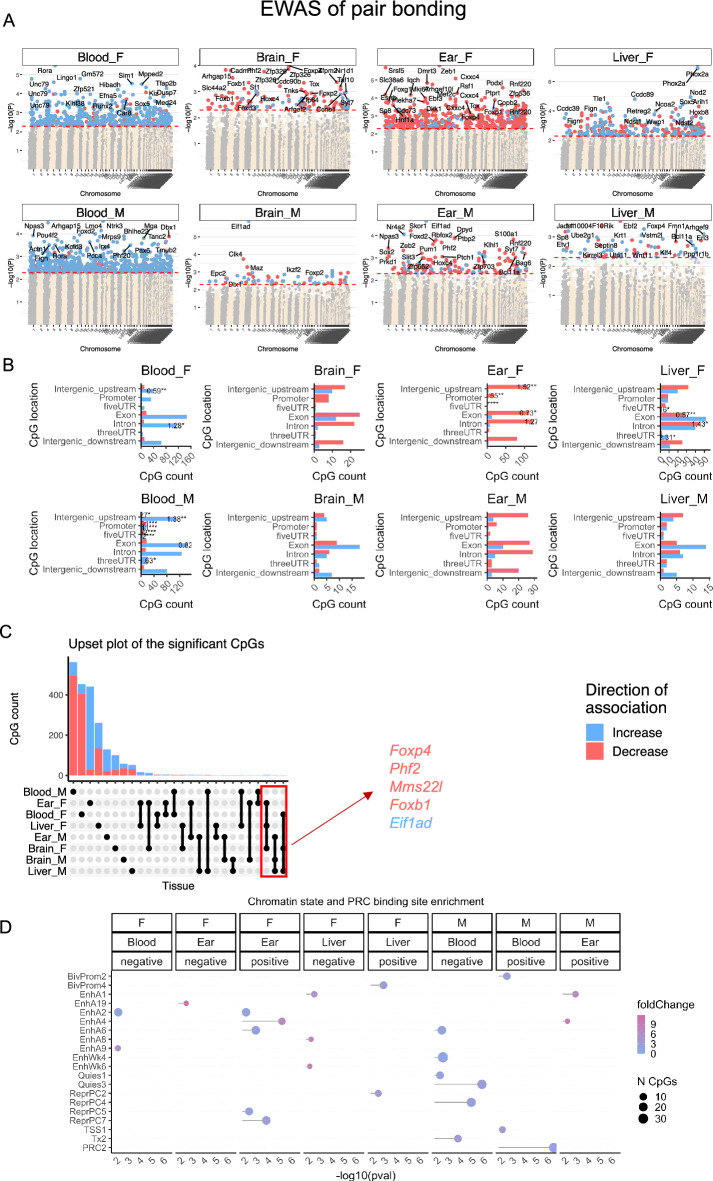
Epigenome-wide association study of pair bonding status from prairie voles (*Microtus ochrogaster*). The association of DNAm and pair bonding was examined by a multivariate regression model for each tissue, adjusting for chronological age as a covariate, and stratified by sex. All voles younger than 0.3 years were sex-naive and were therefore restricted from these analyses. Adult sex-naive females and males (> 0.3 years old) were considered as the reference for comparison with their pair-bonded counterparts. (**A**) Manhattan plots of the EWAS of pair bonding status. Significant CpGs (nominal two-sided *p* < 0.005, red dotted line) are colored in red (methylation increase in pair-bonded animals compared to sex-naive animals) or blue (methylation decrease in pair-bonded animals compared to sex-naive animals). The top 15 CpGs are labeled by the neighboring genes. The x-axis reports genome coordinates based on the alignment of mammalian array probes to the MicOch1.0.100 genome assembly. (**B**) Location of top CpGs in each tissue relative to the closest transcriptional start site. The odds ratio of the proportion changes than the background is reported in for each bar. Fisher exact p values: *p < 0.05, **p  < 0.01, ***p < 0.001, ****p  < 0.0001. The number of selected CpGs: female blood, 465 CpGs; male blood, 569 CpGs; female brain, 119; male brain, 62 CpGs; female ear, 481 CpGs; male ear, 137 CpGs; female liver, 289 CpGs; male liver, 59 CpGs. (**C**) Upset plot representing the overlap of EWAS of pair bonding in different tissues and sexes. Neighboring genes of the overlapping CpGs were labeled in the figure. (**D**) Chromatin state enrichment of the top CpGs related to pair bonding. The nominal two-sided enrichment p-values were calculated with Fisher's hypergeometric test. The universal chromatin states^60^ are based on the stackHMM states in human Hg19 genome. The PRC2 binding is defined by the available ENCODE ChipSeq results of EED, EZH2, and SUZ12 transcriptional factors.

Although pair bonding status does not appear to alter epigenetic age acceleration in prairie vole blood, brain, ear and liver tissues (Fig. 3), we examined pair bonding-associated specific DNAm changes in our EWAS. Pair bonding had a small effect size on DNAm age, so the differences were studied at a nominal significance of *p* < 0.005. The total number of significant differentially methylated CpGs (and most significant hit in pair-bonded voles were: female blood, 465 CpGs, with hypomethylation of the *Rora* intron; male blood, 569 CpGs, with hypomethylation of the *Ntrk3* exon; female brain, 119 CpGs, with hypermethylation of the *Foxp4* intron; male brain, 62 CpGs, with hypomethylation of the *Eif1ad* exon; female ear, 481 CpGs, with hypermethylation of the *Zeb1* exon; male ear, 137 CpGs, with hypomethylation of the *Eif1ad* exon; female liver, 289 CpGs, with hypomethylation upstream of *Phox2a*; male liver, 59 CpGs, with hypermethylation of the *Ebf2* intron (Fig. 5A).

Most of the differentially methylated CpGs in blood showed hypomethylation in pair-bonded animals compared to sex-naive animals, regardless of relationship to transcriptional start sites and sex, with male blood showing a significantly higher percentage of DNAm change than the background (odds ratio of the proportion changes compared to the background and Fisher’s exact *p* values; Fig. 5B). Most of the differentially methylated CpGs in ear tissue showed hypermethylation in pair-bonded animals compared to sex-naive animals, regardless of their relationship to transcriptional start sites and sex, with female ear tissue showing a significantly lower percentage of DNAm change than the background (Fig. 5B). CpGs in brain and liver tissues were distributed in genic and intergenic regions, but not with a higher DNAm percent change than the background (Fig. 5B). CpGs that showed consistent pair-bonding-associated methylation changes across tissues and sexes were identified with an upset plot. The shared pair bonding signatures in at least 3 tissues included hypermethylation in *Foxp4*, *Phf2*, *Mms22l*, and *Foxb1*, and hypomethylation in *Eif1ad* (Fig. 5C). Future replication studies are needed to corroborate these results.

We performed a chromatin state and polycomb repressive complex (PRC) binding site enrichment analysis from our EWAS of pair bonding to characterize chromatin features at which significant pair bonding related CpGs are positioned. Our analysis revealed 7 groups of chromatin states, including bivalent states associated with promoters (BivProm), active and weak enhancers (EnhA, EnhW), quiescent/low states (Quies), polycomb repressed states associated with H3K27me3 (ReprPC), transcription start site (TSS), strong transcription states (Tx), and polycomb repressive complex 2 (PRC2). In blood, pair-bonded males showed a gain of methylation in BivProm2, TSS1, and PRC2 binding sites and a loss of methylation in EnhA6, EnhWk4, Quies1, ReprPC4, and Tx2 (Fig. 5D). Gene level enrichment analysis of CpGs associated with pair bonding was tissue specific (Supplementary Fig. 5).

## Discussion

The development of prairie vole epigenetic clocks described here was based on novel DNA methylation data that were derived from 4 prairie vole tissue types (blood, brain, ear, and liver). We show that the pure pan-tissue vole clock accurately relates chronological age with estimated DNAm age in different prairie vole tissues. This gives us confidence that these clocks will work on new samples from other tissue types as well. The prairie vole DNA methylation profiles reported here represent the most comprehensive dataset thus far of matched single base resolution methylomes across multiple tissues and ages in prairie voles. Future research needs to assess whether DNA methylation lends itself to building biomarkers that measure reproductive fitness potential, potential individual mate quality, and most importantly long-term survivability.

We expect that the availability of these epigenetic clocks will provide a significant boost to the attractiveness of the prairie vole as a biological model in aging research^23,50^. Prairie voles are perhaps best known for their propensity to form human-like socially monogamous pair bonds^12,13^. The human literature has demonstrated overwhelming evidence that there are positive health and longevity benefits associated with healthy supportive marriage partnerships. We used the prairie vole as a model to understand the benefits of paired living. Like humans, pair bonding status is a phenotype that often encompasses several traits and life-history experiences. Pair bonded prairie voles often differ from those that are not bonded in several ways, including age, exposure to offspring, territorial behaviors, and other aspects of life-history that are associated with bonding. Surprisingly, we did not find evidence that tissues from pair-bonded animals age at slower rates than animals that remain single. Our study focused on animals that were approximately double the typical natural lifespan, as most prairie voles only live for between 4 and 7 months in the wild. The oldest animal in our study was 1.3 years old. However, anecdotal evidence from our breeding colony and from other laboratories has suggested that it is possible for prairie voles to live occasionally beyond 3 years. Therefore, we cannot rule out that pair bonding might affect epigenetic age later in life. However, we also note that estimates of the pre-modern civilization lifespan of humans were 30 to 40 years, and the maximum lifespan on record is just over 122 years (about a threefold difference). This difference is comparable to the fourfold difference between the ~ 6 month lifespan in the wild and the ~ 2 year max lifespan noted for most laboratory conditions among prairie voles. This similarity raises interesting questions about possible universal themes across species for the relationship between constraints that ecological pressures place on lifespan and the total potential lifespan a species is capable of.

Since our study only involved captive animals, we are not able to determine whether epigenetic changes explain the substantial difference in life expectancy between captive and wild voles. Others have found that the aging rate in a given species was higher under free-ranging conditions than in captivity^51,52^.

The human literature has found that males are more sensitive to the preserving effects of marriage or comparable long-term social/mating partnerships. Similar male-biased sensitivity among prairie voles has been reported as a result of single-gene DNA methylation associated with early life social experience on later social behavior (specifically social approach)^28^. Nonetheless, we do not find that pair bonding slows overall epigenetic age in prairie voles.

Our EWAS provides evidence linking social monogamous life strategies with alterations in methylation levels of select CpGs. Our EWAS analyses identified CpGs near five genes that were significantly associated with pair bonding in at least three tissue types: *Foxp4*, *Phf2*, *Mms22l*, *Foxb1*, and *Eif1ad*. Although these CpGs are located near certain genes, it is unclear if they influence the gene products, such as mRNA. In short, these CpGs may not correlate with the expression levels of neighboring genes. The relationship between methylation and mRNA is highly complex and varies by cell type. Follow up studies are needed to validate these findings and to elucidate the mechanisms through which they are involved. The CpGs identified by our clock differ from those in the EWAS findings because the EWAS analysis targets individual CpGs related to pair bonding without considering aging. In contrast, our epigenetic clock analysis assesses whether the overall methylation profile in pair-bonded animals indicates an older or younger biological age compared to sex-naive animals.

Beyond their utility, the epigenetic clocks for prairie voles reveal several salient features with importance to the biology of aging. First, the vole pan-tissue clock re-affirms the implication of the human pan-tissue clock that aging might be a coordinated biological process that is harmonized throughout the body. Second, the ability to develop a human-vole pan-tissue clock for relative age attests to the high conservation of the aging process across two evolutionary distant species. A critical step toward crossing the species barrier was the move toward using a mammalian DNA methylation array that profiled 36 thousand probes that were highly conserved across numerous mammalian species^47^. The two human-vole clocks estimate chronological and relative age, respectively. Recently, we presented universal pan-mammalian clocks which indicate that aging can be accurately estimated using DNA methylation profiles, and is evolutionarily conserved and intertwined with developmental processes^40,53^.

## Materials and methods

### Ethics

All experimental procedures were conducted and approved by the Institutional Animal Care and Use Committee (IACUC) of Cornell University (2013-0102) and were in accordance with the guidelines set forth by the National Institutes of Health. All experiments were performed in accordance with relevant ARRIVE guidelines and regulations. No animals were specifically euthanized for this study. Instead, tissue samples were obtained post-mortem from animals that had been euthanized upon completion of separate studies. These tissues were repurposed for the current research. Furthermore, no chemical agents were utilized in the euthanasia process of these animals.

### Prairie vole colony

Male and female prairie voles (*Microtus ochrogaster*) were produced from laboratory-bred colonies at Cornell University, from breeding pairs that were offspring of wild-caught animals captured in Champaign County, Illinois, USA. Voles were weaned and housed with littermates on postnatal day (PND) 21, and then housed with same-sex littermates after PND42-45. All animals received rodent chow (Laboratory Rodent Diet 5001, LabDiet, St. Louis, MO, USA) and water ad libitum and were maintained under standard laboratory conditions (14L:10D cycle, lights on at 08:00, 20 ± 2 °C) in transparent polycarbonate cages (46 × 25 × 15.5 cm) lined with Sani-chip bedding and provided nesting material.

### Prairie vole tissue sample collection

Blood, brain, ear, and liver samples from the Cornell University prairie vole colony were collected from 96 male and female prairie voles at various life stages: neonatal (< 1 month old), sub-adult (2–4 months old), mature adult (4–10 months old), and middle-aged/old adult (> 10 months old). The term “pair-bonded” is typically used to describe prairie voles that display a preference for their partner over an unfamiliar stranger after cohabitating and mating with their partner for between 24 h to 2 weeks. These studies typically assess central and peripheral changes at an endpoint right before birth occurs or prevent pregnancy altogether to eliminate pregnancy or parenting as confounding factors. Our study includes voles that never experienced mating and voles that experienced several life history events with their mating partner, such as mating and cohabitating for a very long extended period, and birthing and rearing at least three litters of offspring. Therefore, it is important to note that we use the term “pair-bonded” as an oversimplified term to capture these major differences in life-history not experienced by sex-naive animals in this study. Animals were euthanized via rapid decapitation, their tissues rapidly extracted and frozen on dry ice before being stored at − 80 °C until further processing for genomic DNA extraction. Brains were coronally sectioned and brain regions from the pair bonding circuit (PBC) were micro-dissected and pooled for each animal. The PBC brain regions included the prefrontal cortex, nucleus accumbens, lateral septum, ventral pallidum, and medial amygdala, and ventral tegmental area^54^. Genomic DNA was isolated and purified using the phenol–chloroform extraction and ethanol precipitation method for the brain, ear, and liver samples. Genomic DNA from blood samples was extracted using the DNeasy Blood & Tissue Kit (Cat. # 69504, Qiagen, Germantown, MD). A total of 336 tissue samples (48 blood, 96 brain, 96 ear, and 96 liver) were collected and processed for DNA methylation analysis. One animal was removed from the study due to a mismatch with the reported sex and our DNA methylation-based sex estimator.

### Human tissue samples

To build the human-vole clock, we analyzed previously generated methylation data from 1366 human tissue samples (adipose, blood, bone marrow, dermis, epidermis, heart, keratinocytes, fibroblasts, kidney, liver, lung, lymph node, muscle, pituitary, skin, spleen) from individuals whose ages ranged from 0 to 93. These publicly available data have been described in other articles^40,55^.

### DNA methylation data

We generated DNA methylation data using the custom Illumina chip "HorvathMammalMethylChip40" following a previously described procedure^47^. The mammalian methylation array is attractive because it provides very high coverage of highly conserved CpGs in mammals (up to 36 k CpGs per species). Two thousand out of 38 k probes were selected based on their utility for human biomarker studies: these CpGs, which were previously implemented in human Illumina Infinium arrays (EPIC, 450 K) were selected due to their relevance for estimating age, blood cell counts, or the proportion of neurons in brain tissue. The remaining 35,988 probes were chosen to assess cytosine DNA methylation levels in mammalian species. The subset of species for each probe is provided in the chip manifest file which can be found at Gene Expression Omnibus (GEO) platform GPL28271. The SeSaMe normalization method was used to define beta values for each probe^56^.

### Penalized regression models

We have implemented R software functions and related tutorials in our MammalMethylClock R package, which provides tools for constructing and assessing epigenetic clocks^57^. Details on the clocks (CpGs, genome coordinates) and additional R software code are provided in the Supplement. Penalized regression models were created with glmnet^58^. We investigated models produced by both “elastic net” regression (alpha = 0.5). The optimal penalty parameters in all cases were determined automatically by using a tenfold internal cross-validation (cv.glmnet) on the training set. By definition, the alpha value for the elastic net regression was set to 0.5 (midpoint between Ridge and Lasso type regression) and was not optimized for model performance.

We performed a cross-validation scheme for arriving at unbiased (or at least less biased) estimates of the accuracy of the different DNAm based age estimators. One type consisted of leaving out a single sample (LOOCV) from the regression, predicting an age for that sample, and iterating over all samples. A critical step is the transformation of chronological age (the dependent variable). While no transformation was used for the pan-tissue clock for voles, we did use a log linear transformation for the dual species clock of absolute age.

To introduce biological meaning into age estimates of voles and humans that have very different lifespan; as well as to overcome the inevitable skewing due to unequal distribution of data points from voles and humans across age range, relative age estimation was made using the formula: Relative age = Age/maxLifespan where the maximum lifespan for the two species was chosen from the *AnAge* database^59^. Thus, the maximum age of voles and humans was set at 5.3 and 122.5 years, respectively. Age acceleration, defined as increased epigenetic age relative to chronological or relative age, was calculated by taking the residual term of the multivariate regression model for each clock. A positive residual term indicates faster epigenetic aging while a negative residual term indicates slower epigenetic aging.

### Epigenome wide association studies of age

EWAS was performed in each tissue separately using the R function "standardScreeningNumericTrait" from the "WGCNA" R package. Next the results were combined across tissues using Stouffer's meta-analysis method.

### Chromatin statement enrichment analysis

Chromatin state enrichment of the top CpGs related to pair bonding. The nominal two-sided enrichment p-values were calculated with Fisher's hypergeometric test. The background is limited to the CpGs that are annotated for chromatin states and mapped to Prairie vole. The universal chromatin states^60^ are based on the stackHMM states in human Hg19 genome. The PRC2 binding is defined by the available ENCODE ChIP-Seq results of EED, EZH2, and SUZ12 transcriptional factors.

### Supplementary Information

Supplementary Information 1.

Supplementary Information 2.

Supplementary Information 3.

Supplementary Information 4.

Supplementary Information 5.

## Data Availability

The individual-level data from the Mammalian Methylation Consortium can be accessed from several online locations. All data from the Mammalian Methylation Consortium are posted on Gene Expression Omnibus (complete dataset, GSE223748). The mammalian data can also be downloaded from the Clock Foundation webpage: https://clockfoundation.org/MammalianMethylationConsortium. The mammalian methylation array is available through the non-profit Epigenetic Clock Development Foundation (https://clockfoundation.org/). The manifest file of the mammalian array and genome annotations of CpG sites can be found on Zenodo (10.5281/zenodo.7574747). Additional annotations can be found on our Github page: https://github.com/shorvath/MammalianMethylationConsortium/tree/v2.0.0.

## Code availability

The epigenetic clocks are implemented in the R software code in the Supplementary data file and in the MammalMethylClock R package^58^.
