# Supplementary material for: Epigenetic aging studies of pair bonding in prairie voles
Source: Sci Rep. 2024 Jul 29;14:17439. doi: 10.1038/s41598-024-67641-2 (PMC11286801; doi:10.1038/s41598-024-67641-2)
Supplement: Supplementary file 3 — Supplementary Information 3. [file 41598_2024_67641_MOESM3_ESM.docx]

**Supplementary Information**

**for “Epigenetic aging studies of pair bonding in prairie voles” by Sailer et al**

Contents

Supplementary Figures.

Technical Details surrounding the DNAm age estimator.

**Supplementary Figure 1. Unsupervised hierarchical clustering of tissue samples from prairie voles**. Average linkage hierarchical clustering based on the inter-array correlation coefficient (Pearson correlation). The first color band is based on cutting the branches at a height cut-off of 0.04. Note that the branch colors correspond to tissue (second color band): blue=brain, brown=ear, turquoise=blood, yellow=liver. The brain samples derive from the pooled pair bonding circuit (PBC) brain region. The third color band visualizes pair bonding status: red=pair bonded, black=sex naïve. Pair bonding status does not seem to correspond to distinct clusters. Fourth color band visualizes age. Fifth color band visualizes sex: light blue=male, pink=female.


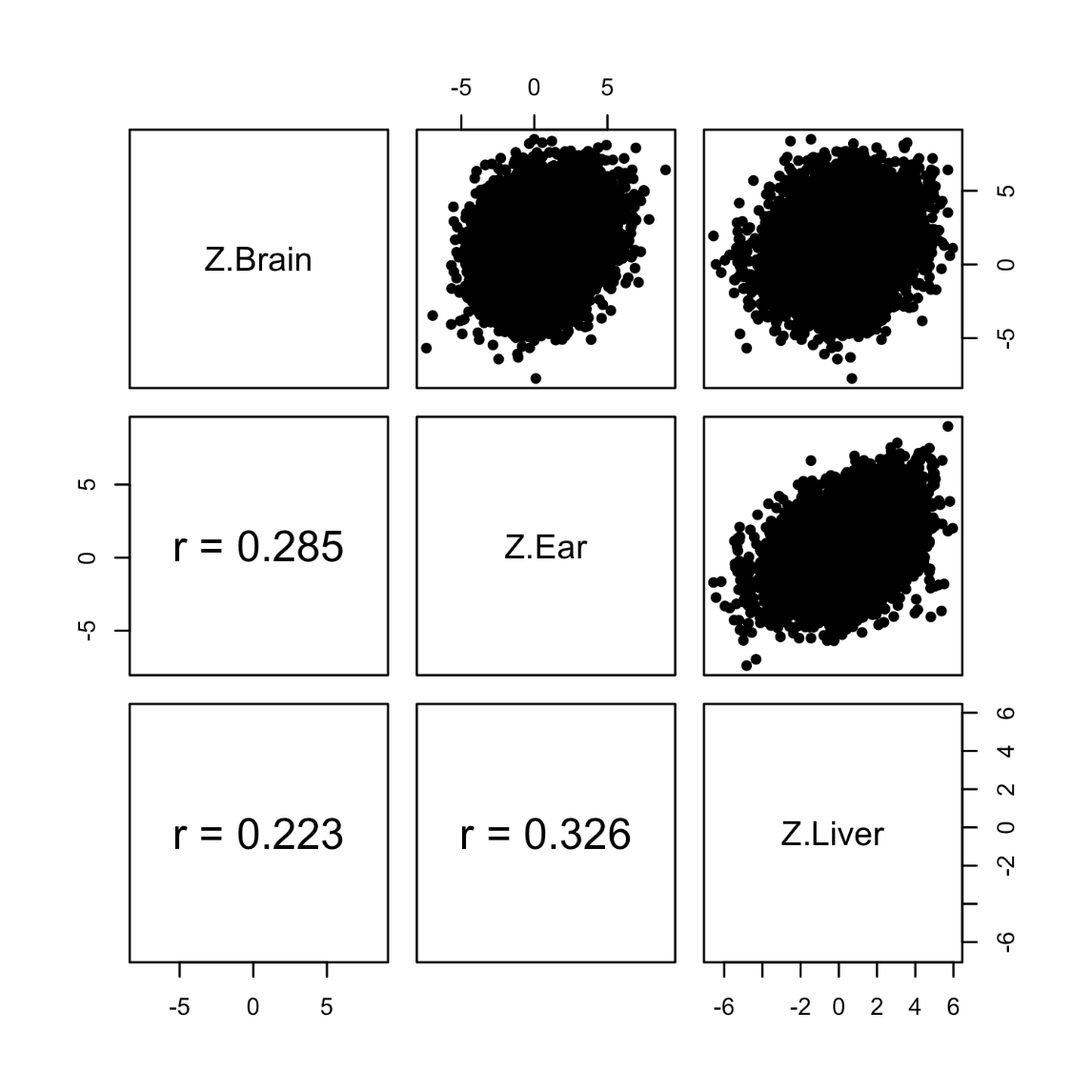


**Supplementary Figure 2. Epigenome wide association study of correlation in three different tissues**. Each dot corresponds to a CpG. Z statistics for a correlation test of age in brain, ear, liver.

**Supplementary Figure 3: Enrichment Analysis of Top Age-Associated CpGs.** Enrichment was assessed using the genomic region enrichment annotation tool (GREAT) against a human Hg19 reference ^1^. Gene-level enrichment was limited to the 22,264 background probes that align with identical genes in the prairie vole genome. Analyses incorporated datasets from gene ontology, mouse phenotypes, promoter motifs, and MsigDB Perturbation, which encompasses expression signatures of genetic perturbations as cataloged in the GSEA database. Significance was determined at a nominal two-sided p < 10^-3^, highlighting only the top terms from each EWAS finding.

**Supplementary Figure 4: Enrichment in Chromatin States of Age-Associated CpGs. Enrichment p-values were derived using a hypergeometric test.** Chromatin states refer to those defined by stackHMM in the human Hg19 genome, as detailed in Vu and Ernst ^2^. PRC2 binding sites were identified using ENCODE ChipSeq data for the transcription factors EED, EZH2, and SUZ12.


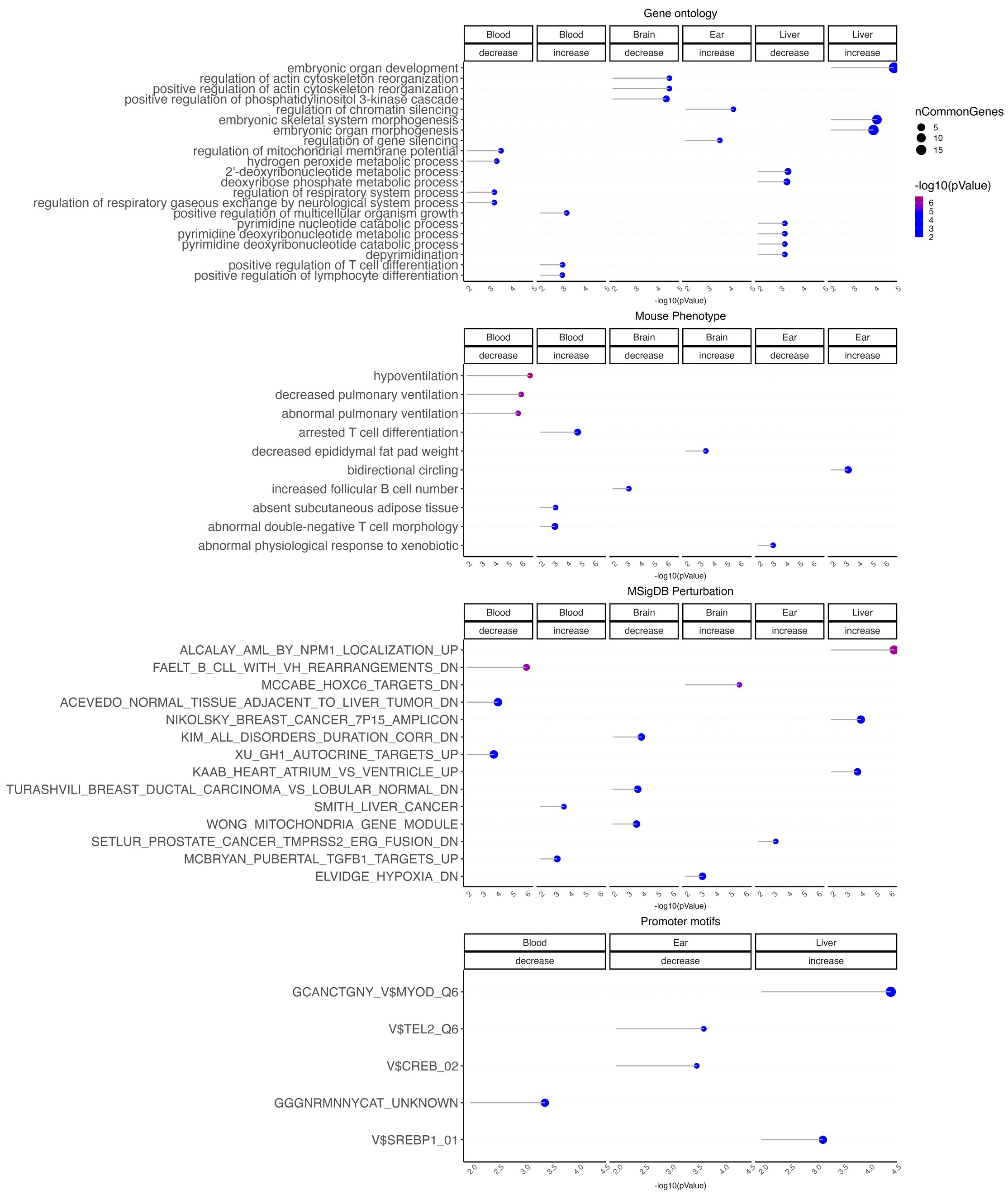


**Supplementary Figure 5: Top CpGs Associated with Pair Bonding:** Enrichment Analysis. This analysis utilized the genomic region enrichment annotation tool (GREAT) with gene-level enrichment against the human Hg19 reference ^1^. Background probes were restricted to the 22,264 that correspond to identical genes in the prairie vole genome. Data sources included gene ontology, mouse phenotypes, promoter motifs, and the MsigDB Perturbation database, which houses expression signatures of genetic perturbations from the GSEA database. Results were considered significant at a nominal (uncorrected) p < 10^-3^, highlighting only the most pertinent terms from each EWAS finding.

**Technical Details surrounding the DNAm age estimator**

**Statistical methods used for building the clocks**

The epigenetic clocks were used by employing a single elastic net regression model analysis (R function glmnet). We use used Leave-one-out analysis (LOO) using a single lambda value. We chose the following parameters for the glmnet R function (Alpha: 0.5, CV Fold: 10, Lambda choice for Clock: 1 standard error above minimum CV-MSE). The coefficient values can be found in the Supplementary Table 1.

**Covariates and coefficient values of the voleclocks**

1. The volepan tissue clock is based on 241 CpGs whose coefficient values are specified in the column "VoleMultiTissue". Age transformation=identity, i.e. F(Age)=Age
2. The vole blood tissue clock is based on 26 CpGs whose coefficient values are specified in the column "Coef.VoleBlood". Age transformation=identity, i.e. F(Age)=Age
3. The vole Liver tissue clock is based on 53 CpGs whose coefficient values are specified in the column "Coef.VoleLiver". Age transformation=identity, i.e. F(Age)=Age
4. The vole Brain tissue clock is based on 35 CpGs whose coefficient values are specified in the column "Coef.VoleBrain". Age transformation=identity, i.e. F(Age)=Age
5. The Human Vole clock for chronological age is based on 622 CpGs whose coefficient values are specified in the column "Coef.HumanVoleLogLinearAge". Age transformation=log-linear described below.
6. The final human voleclock for relative age is based on 656 CpGs whose coefficient values are specified in the column "Coef.HumanVoleRelativeAge". Age transformation: relative age. i.e. F(Age)=Age/maxLifespan. Human max lifespan =122.5 years. According to the data base "anAge" [3], the maximum lifespans are as follows: 5.3 years for prairie voles. While the maximum lifespan estimates may be debatable, our clock is quite robust with respect to different choices of this mathematical parameter. Similarly accurate clocks could be constructed with different parameter choices.
7. The vole clock for pair bonded animals consists of 112 CpGs. VoleClockForPairBonded
8. The vole clock for sex niave animals consists of 166 CpGs. VoleClockForSexNaive

**General description of age transformation**

The human-*Vole* clocks for chronological age used log linear transformations that are similar to those employed for the HUMAN pan tissue (Horvath 2013) [6].

An elastic net regression model (implemented in the glmnet R function) was used to regress a transformed version of age on the beta values in the training data. The glmnet function requires the user to specify two parameters (alpha and beta). Since I used an elastic net predictor, alpha was set to 0.5. But the lambda value of was chosen by applying a 10-fold cross validation to the training data (via the R function cv.glmnet).

The elastic net regression results in a linear regression model whose coefficients b_0_, b_1_, . . . , relate to transformed age as follows
*F*(chronological age)=*b*_0_*+b*_1_*CpG*_1_*+ . . . +b*_p_*CpG*_p_+error

Note that the intercept term is denoted by b_0_. The coefficient values can be found in the attached Excel file.

Based, on the coefficient values from the regression model, DNAmAge is estimated as follows
*DNAm*Age=$F^{-1}$(*b*_0_*+b*_1_*CpG*_1_*+ . . . +b*_p_*CpG*_p_)

where $F^{-1}\left( y \right)$ denotes the mathematical inverse of the function F(.). Thus, the regression model can be used to predict to transformed age value by simply plugging the beta values of the selected CpGs into the formula.

To use this transformation to predict Age on *new samples*, one needs to use the *inverse* transformation, F^-1^(y), given by

$$F^{-1}\left( y \right)= \left\{ \begin{aligned} \begin{aligned} \begin{aligned} \left( 1.5*A+G \right)*\text{exp}\left( y \right)-G, for y\leq0 \\ \left( 1.5*A+G \right)*y+1.5*A, for y\geq0 \end{aligned} \end{aligned} \end{aligned} \right.$$

where $A$ refers to age at sexual maturity (in years) and $G$ refers to gestation time (in years). For predicting age, apply the inverse transformation to coefficient-weighted sum. That is,

$$DNAmAge=F^{-1}\left( x*\beta\right)$$

where $\beta$ is the vector of coefficients and $x$ is the vector of methylation values, with an intercept term.

For the human-vole clocks we used the following average ages at sexual maturity (in units of years): 13.5 years for humans and 0.128767123 years for vole. In addition, we used the following average gestation times (in units of years): 0.767123288 years for humans and 0.063013699 years for vole.

## **The DNAm Age estimate is estimated in two steps.**

First, one forms a weighted linear combination of the CpGs whose details can be found in Table

The table reports the probe identifier (cg number) used in the custom Infinium array (HorvathMammalMethylChip40) . The weights used in this linear combination are specified in the respective column entitled "Coef.".

The formula assumes that the DNA methylation data measure "beta" values but the formula could be adapted to other ways of generating DNA methylation data.

References

1 McLean, C. Y. *et al.* GREAT improves functional interpretation of cis-regulatory regions. *Nat Biotechnol* **28** (2010). <https://doi.org:10.1038/nbt.1630>

2 Vu H, Ernst J. Universal annotation of the human genome through integration of over a thousand epigenomic datasets. Genome Biol. 2022;23(1):9. Published 2022 Jan 6. doi:10.1186/s13059-021-02572-z
